# Effect of Time-Delayed Feedback on the Interaction of a Dimer System with its Environment

**DOI:** 10.1038/s41598-017-15185-z

**Published:** 2017-11-13

**Authors:** M. Farhat, S. Kais, F. H. Alharbi

**Affiliations:** 1 Qatar Environment and Energy Research Institute (QEERI), Hamad Bin Khalifa University, Qatar Foundation, Doha, Qatar; 2 Department of Chemistry, Department of Physics and Birck Nanotechnology Center, Purdue University, West Lafayette, IN 47907 USA; 3 College of Science and Engineering, Hamad Bin Khalifa University, Doha, Qatar

## Abstract

In this work, we report modeling of non-Markovian open quantum systems, consisting of an excitonic dimer that displays memory effect due to time delayed interaction with its environment. We, indeed investigate the effect of these time delays on quantum coherence and excitation dynamical behavior in the time domain generally considered for photosynthetic experiments (few hundred femtoseconds). In particular, we show that the coherence is maintained for periods proportional to time delays. Additionally, if delay is taken into account, coupling to the environment can be tuned to lower values, unlike in previous studies. This kind of intriguing effect can, therefore, when generalized to complete systems, permit more control on the experimental parameters, which may lead to more accurate description of the photosynthetic energy transfer functioning and subsequent applications in artificial photovoltaic research.

## Introduction

Photosynthesis is the main mechanism by which solar energy is converted into chemical and biological energy on earth. The captured sunlight (electronic excitation) is transferred from the antenna system to the reaction center, by transporting the electronic excitation that can be used for the charge separation initiating a bunch of biochemical processes^1^. This light harvesting and exciton transport by the photosynthetic protein complexes occurs with a surprisingly high efficiency. It is believed that this is quantum mechanical effect and should be accordingly explained by models based on quantum theory^2–11^. In fact, some of the states of molecular vibration of the chromophores facilitate the transfer of energy during the process of photosynthesis and contribute to its effectiveness^11^. Thus, for example, when two chromophores vibrate, it happens that certain energies associated with these collective vibrations of the two molecules are such that they correspond to transitions between two levels of electronic energy of the molecules. A resonance phenomenon occurs and a transfer of energy flows between the two chromophores^8^.

Several studies have reported the quantum coherence effect in excitonic transport in Fenna-Matthews-Olson (FMO) complexes. This was even experimentally proved in the seminal study of Engel and Scholes in 2007^2^ using 2D Fourier transform electronic spectroscopy^12,13^. Generally, the community concentrated on studying these effects on the FMO complex, that consists of Bacteriochlorophyll (BChl) pigments bound to a protein scaffold^14^. FMO is generally found in green sulfur bacteria and is a simple system that can be adequately described theoretically and numerically. This complex is a trimer, where each monomer consists of eight BChl molecules, where the 8*th* BChl is though weakly bound^15^. The interest fueled among the community on FMO is justified by its well-documented structure, as well as its solubility in water, facilitating thus experimental uses^16^.

Soon after, several theoretical studies have been proposed in order to explain these somewhat counter-intuitive and unexpected features, i.e. highly efficient energy transfer at physiological temperatures. In fact, one would expect that a very fast decoherence due to strong interaction between the system and bath of oscillators occurs, and therefore leads to energy and phase relaxation, thus allowing for downhill directed transfer of energy^17,18^. However, experimental works (seminal work of Engel^2^) showed that decoherence times in FMO very much exceeded expected values calculated using traditional line-widths of electronic transitions^19^. This evidence means that the coupling to the vibrational bath must be more important than anticipated, in particular the vibrational modes with frequencies near the electronic frequency gaps between the BChl sites (to accurately model the energy transfer dynamics in the FMO complex). To this purpose, many theoretical studies used different methods to study the excitonic dynamics, e.g. hierarchy equation of motion^20–22^, multi-configuration TD Hartree^17^, Redfield modified equations^23–25^, path integral approach^26^.

Unlike Markovian quantum systems, where deriving memory-less equations of motion is a feasible task^27–29^, the study of open quantum systems with random memory (or quantum feedback) effect is more elusive, because of the absence of systematic tools independent of the system-environment specific interaction^30,31^. In this context, Carmichael, Gardiner, and Sokolov, independently developed the theory of cascaded quantum open systems^32–34^ where typically the output from one system is fed into another one. Conversely, coherent quantum feedback is completely different in the sense that it is not mediated by a sensor or measurement whose results are supplied to the quantum system, but rather it is a consequence of the dynamics of the system-bath coupling^35^. In this context Grimsmo *et al*. have proposed a control scheme designed to reduce the time a damped, driven system takes to reach a steady state^36,37^.

In this report, we utilize the modeling where delay is introduced to quantum open systems (bosonic and fermionic, alike) as in ref.^38^ and we propose to study a general example of a dimer, i.e. a two-level system basically (that can be used as a benchmark for describing a system of two specific BChl, as was done for example in refs^39–41^). and the population beating due to feedback in the interaction with a bath of phonons. We believe that such memory effect (non-Markovianity) can have a role in the excitonic dynamics of more complex FMO systems and that this simple model shows that taking into account this effect, one is able to reduce the coupling to vibrations (in coherence with the expected line-widths). We believe also that a more complete treatment taking into account this feedback can help in shedding new light towards the understanding of efficient energy transfer in photosynthetic organisms and to be able to propose artificial devices based on this concept^10,42,43^.

The outline of this manuscript is the following, in the first section we derive the theoretical model describing delay in the interaction between a two-level system and its environment (bath of phonons). In next section, we use this simplified model of dimer to study the dynamics of the populations. We finally, investigate the role of the delay $\tau$ in the beating effect and show how this can help in reducing the high coupling to environment.

## Theoretical modeling

Frenkel exciton Hamiltonian is generally used to describe excitonic dynamics, that consists of *N* sites, each having the excitation energy $\hslash {{\rm{\Omega }}}_{n}$. This system is embedded in a reservoir (bath) of phonons (collection of harmonic oscillators) of energies $\hslash {\omega }_{m,\xi }$ (where each site is assumed to be associated to its own bath).

### System Hamiltonian and approximations

The Hamiltonian that describes the complete dynamics of Frenkel excitons is given in the form^11^

1 $$H={H}_{S}+{H}_{SE}+{H}_{E},$$

where $HS=\sum _{n,n^{\prime} }\,(\hslash {{\rm{\Omega }}}_{n}{\delta }_{n,n^{\prime} }+{V}_{n,n^{\prime} }(t)){a}_{n}^{\dagger }{a}_{n^{\prime} }$, ${H}_{E}=\sum _{m,\xi }\hslash {\omega }_{m,\xi }{b}_{m,\xi }^{\dagger }{b}_{m,\xi }$, and ${H}_{SE}$ denote the system, the environment, and the interaction Hamiltonian, respectively. Equation (1) can be re-cast in the more convenient form

2 $$H={H}_{0}+{H}_{A}+{H}_{SE},$$

where we have defined

3 $${H}_{0}=\sum _{n}\hslash {{\rm{\Omega }}}_{n}{a}_{n}^{\dagger }{a}_{n}+\sum _{m}\sum _{\xi }\hslash {\omega }_{m,\xi }{b}_{m,\xi }^{\dagger }{b}_{m,\xi },$$

where ${a}_{n}$ $({a}_{n}^{\dagger })$ and ${b}_{m,\xi }$ $({b}_{m,\xi }^{\dagger })$ denote annihilation (creation) operators of an excitation of the system and reservoir, respectively. The time-dependent Hamiltonian ${H}_{A}(t)$ represents the coupling between the neighboring sites, i.e. the detuning from the central frequencies ${{\rm{\Omega }}}_{n}$

4 $${H}_{A}(t)=\sum _{n,n^{\prime} }\,{V}_{n,n^{\prime} }(t){a}_{n}^{\dagger }{a}_{n^{\prime} }.$$

The system-environment (sites-bath) interact via the Hamiltonian^4^

5 $${H}_{SE}=\sum _{n}\sum _{m,\xi }({\kappa }_{n,m,\xi }{\sigma }^{+}{b}_{m,\xi }+{\kappa }_{n,m,\xi }^{\ast }{\sigma }^{-}{b}_{m,\xi }^{\dagger }),$$

where each site is supposed to to be coupled to an independent bath of harmonic oscillators. ${\sigma }^{+}$ and ${\sigma }^{-}$ are the pseudo-spin operators of a two-level system and are related to the electronic operators as ${\sigma }^{+,-}={a}_{1,2}^{\dagger }{a}_{2,1}$. The constants ${\kappa }_{n,m,\xi }$ denote the coupling strength between electronic excitation *n* and *m* bosonic bath modes of momenta $\xi$. The coupling is assumed to be linear in the coordinates of the harmonic oscillator. Additionally, the total excitation is conserved.

The initial state of the total system (sites-bath) is given in terms of the decoupled density matrix^29^

6 $$\rho (0)={\rho }_{S}\otimes {\rho }_{E}={p}_{0}|0\rangle \langle 0|+(1-{p}_{0})|\psi (0)\rangle \langle \psi (0)|.$$

At time *t* and due to excitation number conservation, the sate $\psi (t)$ can be expanded as^29^

7 $$\psi (t)={c}_{0}|0\rangle +\sum _{n}{c}_{n}(t){a}_{n}^{\dagger }|0\rangle +\sum _{m,\xi }{d}_{m,\xi }(t){b}_{m,\xi }^{\dagger }|0\rangle .$$

Substituting Eq. (7) in the Schrödinger equation, one can get the differential equation for the amplitudes of the system^29^

8 $$\frac{d}{dt}{c}_{j}(t)=-i\sum _{l}{{\rm{\Delta }}}_{j,l}(t){c}_{l}(t)-\sum _{l}{\int }_{0}^{t}dt^{\prime} {F}_{j,l}(t,t^{\prime} ){c}_{l}(t^{\prime} ),$$

with $${{\rm{\Delta }}}_{j,l}(t)=\langle 0|{a}_{j}{H}_{A}(t){a}_{l}^{\dagger }|0\rangle $$ and

9 $${F}_{j,l}(t,t^{\prime} )={e}^{i({{\rm{\Omega }}}_{j}t-{{\rm{\Omega }}}_{l}t^{\prime} )}\sum _{m}{\int }_{-{\rm{\infty }}}^{{\rm{\infty }}}d\omega {J}_{m,j,l}(\omega ){e}^{-i\omega (t-t^{\prime} )},$$

where we have defined the spectral densities, in their most general form ${J}_{m,j,l}(\omega )={\sum }_{\xi }{\kappa }_{j,m,\xi }{\kappa }_{l,m,\xi }^{\ast }\delta (\omega -{\omega }_{m,\xi })$.

### System and discrete delay

**Figure 1:**
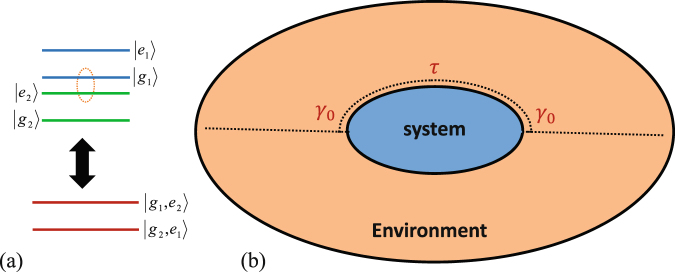
(**a**) The two interacting TLS (top) and their effective modeling (bottom) as a single two-level system, with |*e*_1_〉, |*g*_1_〉 and |*e*_2_〉, |*g*_2_〉 the excited and ground states of the two TLS, respectively. (**b**) Scheme of the interaction between the system and the environment, showing the delay *τ* and the interaction strength constant *γ*_0_.

In particular, we consider in this study a simplified system, i.e. a system consisting of two sites. Each of these sites can be approximated by a two-level system (as depicted in Fig. 1(a))^39^. And since the intensity of incident light excitation is generally weak, one can further approximate these two sub-systems by an effective other two-level system (see Fig. 1(a))^41^ coupled to a bosonic environment at two spatial different locations (as schematically depicted in Fig. 1(b)), forming a coherent feedback loop with time delay $\tau$ and time domain interval *M* ^38^. The Hamiltonian of the environment is expressed as ${H}_{E}={\sum }_{\xi =-\infty }^{\infty }\hslash {\omega }_{\xi }\mathrm{(1/2}+{b}_{\xi }^{\dagger }{b}_{\xi })$, leading to the total free Hamiltonian for the system and environment

10 $${H}_{0}=\frac{1}{2}\hslash {{\rm{\Omega }}}_{0}{a}_{n}^{\dagger }{a}_{n}+\sum _{\xi =-{\rm{\infty }}}^{{\rm{\infty }}}\hslash {\omega }_{\xi }{b}_{\xi }^{\dagger }{b}_{\xi },$$

where the bosonic environment operators satisfy the usual commutation relations $[{b}_{\xi },{b}_{{\xi }^{\text{'}}}^{\dagger }]={\delta }_{\xi {\xi }^{\text{'}}}$ and where we neglect the zero-point energy term in the free system Hamiltonian. The coupling between the system and the environment is assumed to be at two spatial locations, and the interaction Hamiltonian is given in the framework of the rotating wave approximation by

11 $${H}_{SE}=\sum _{\xi }{\kappa }_{\xi }({b}_{\xi }{\sigma }^{+}+{b}_{\xi }^{\dagger }{\sigma }^{-}),$$

with ${\sigma }^{+}$ and ${\sigma }^{-}$ the pseudo-spin operators of a two-level system^38^. The coupling coefficients in this scenario (depicted in Fig. 1(b)) ${\kappa }_{\xi }=\sqrt{2{\gamma }_{0}/M}\,\cos (\bar{\xi }({\omega }_{\xi }\tau /2+{\varphi }_{0}))$, with $\overline{\xi }$ denoting the sign of $\xi$ and *M* a time parameter describing the time interval that we will take the limit to infinity at the end, and ${\gamma }_{0}$ the coupling parameter. The spectral density $J(\omega )={\sum }_{\xi }|{\kappa }_{\xi }{|}^{2}\delta (\omega -{\omega }_{\xi })$, and in the continuum limit, one get

12 $$J(\omega )=\frac{{\gamma }_{0}}{\pi }(1+\,\cos (\omega \tau +\varphi )),$$

for $\omega  > 0$, and with $\varphi =2{\varphi }_{0}$. Substituting Eq. (12) in Eq. (9), one further get the dissipation kernel of the system, using a reverse Fourier transform, i.e.

13 $$f(t-t^{\prime} )={\gamma }_{0}({e}^{i\varphi }\delta (t-\tau -t^{\prime} )+2\delta (t-t^{\prime} )+{e}^{-i\varphi }\delta (t+\tau -t^{\prime} )).$$

Using this form for the dissipation kernel, it is straightforward to show that using Eq. (8), the time evolution of the system amplitude obeys the delay differential equation

14 $$\frac{d}{dt}c(t)=-i{\delta }_{0}c(t)\,-\,{\gamma }_{0}c(t)\,-\,\theta (t-\tau ){\gamma }_{0}{e}^{-i\varphi }c(t\,-\,\tau ),$$

with the right-side parameters normalized with respect to the Planck’s constant *ħ*, and $\theta (t)$ the Heaviside function. Also the detuning term ${\delta }_{0}$ is assumed to be zero, i.e. the coupling between sites occurs only through their mutual coupling to the environment.

## Results

### Population beating and decoherence in the dimer system

We are now in a position to discuss the role played by time delay in the interaction between the two-level system (TLS) and the environment on the evolution of the populations dynamics and coherence, after initial excitation of the first site. Equation (14) can be solved analytically, by Fourier transforming it (in the frequency-domain) and extracting the eigenvalues. This class of differential equations is also known to possess an infinite number of complex eigenvalues, leading to some stability issues related to stiffness^44,45^. Here we solve it numerically using the delay differential equation solver of Matlab^46^, in order to obtain the relation between different parameters of the TLS and amplitudes.

**Figure 2:**
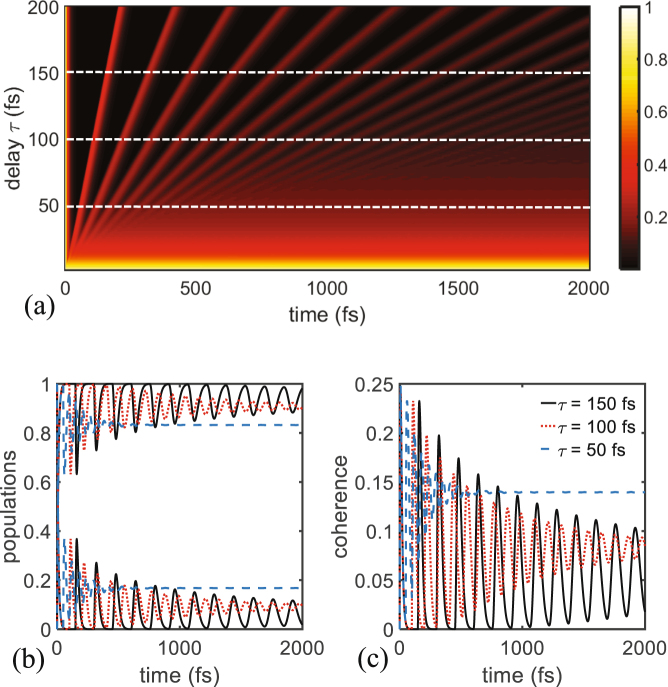
(**a**) Two-dimensional plot showing the variation of the population of the donor site as function of time *t* and delay time $\tau$ in unit of femtoseconds. (**b**) Populations of the donor and acceptor for three specific delay times, corresponding to the white-dashed lines in (**a**). (**c**) Same as in (**b**) but here for the coherence between both sites, in the time interval 0–2000 fs. The numerical values of the other parameters are ${\gamma }_{0}=530$ cm^−1^ and $\varphi =\pi$.

Figure 2(a) plots the population of the donor site versus time lag $\tau$ and evolution time *t*, in the femtosecond range. One can see from this figure that population of site 1 undergoes several quantum oscillations (beating) in the time domain of study (1–2000 fs). The domain of these oscillations clearly increase as well as their period, with increasing values of $\tau$. The dashed white lines in Fig. 2(a) denote three specific values of the delay $\tau$, that are 50, 100, and 150 fs, and are plotted in Fig. 2(b,c) for the populations and decoherences, respectively. Here the decoherence is defined in the usual way as the off-diagonal term of the density matrix, or equivalently $|{c}_{1}{(t)}^{\ast }{c}_{2}(t)|$, with ${c}_{1}(t)$ and ${c}_{2}(t)$ the components of the total amplitude *c*, appearing in Eq. (14). For the smallest values of $\tau$ depicted at the bottom of Fig. 2(a) with the yellow color, the population has almost constant values close to 1, meaning that there is nearly no coupling between the sites. For appreciably higher values of $\tau$, one can see in Fig. 2(b) that the dynamics is considerably different, with the beatings becoming more pronounced as $\tau$ is increased from 50 to 150 fs. Similar effect can be observed in Fig. 2(c) for the decoherence rates where the quantum mechanical beatings live clearly longer due to higher delays that increase the interaction between the two sites via coherent feedback with the phononic bath environment. These oscillations can be interpreted in the following way: energy is transferred from the system into the environment during the positive regions of the decoherence and later re-emitted back towards the system at the negative regime of the decoherence rates^47^.

**Figure 3:**
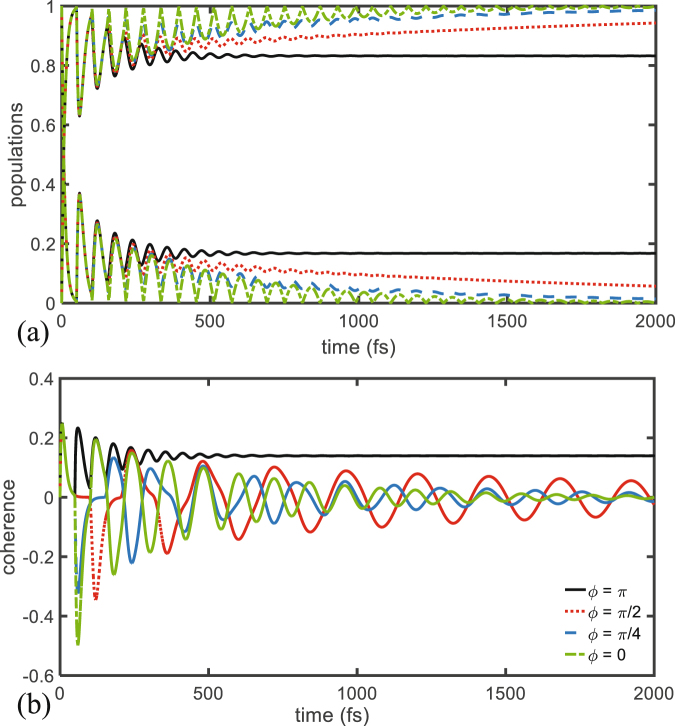
(**a**) Populations and (**b**) coherences for the dimer system with delay time $\tau =50$ fs, coupling to the environment ${\gamma }_{0}=530$ cm^−1^ for different values of the phase parameter $\varphi$.

It should be mentioned that if the TLS reaches a steady state in which $c(t\,-\,\tau )=c(t)$ the decoupling will persist, and excitations can become trapped in the system-loop subsystem. This effect can be seen in Fig. 3 where populations and coherences are depicted for different values of the phase $\varphi$. In particular, for $\varphi =\pi$, destructive interference occurs and it corresponds to negative feedback effect, leading to stabilization of the donor site in a state of partial excitation (solid black curve in Fig. 3). In fact it is possible to show that the amplitude $c(t)$ solution of Eq. (14) tends to $\mathrm{1/(1}+{\gamma }_{0}\tau )$ if $1+{e}^{i\varphi }=0$ and to zero otherwise, when *t* goes to $\infty$. The other extreme scenario occurs thus when $\varphi =0$, i.e. for constructive interference, corresponding to positive feedback that increases the decay rate of the donor site and ultimately leads to its de-excitation as can be seen in Fig. 3 for the dashed green curve. The two other plots correspond to intermediate values of the phase, i.e. $\varphi =\pi \mathrm{/4}$ and $\pi \mathrm{/2}$, and one can see that in the interval 1–2000 fs, the decay of the population of site 1 is much lower than in the case of constructive interference.

These results show the importance of taking into account the role played by delay in the interaction of a TLS with phononic environment, in contrast to previous studies that only considered the effect of non-Markovianity^40^, the environment timescale^48^, and the temperature^49^.

**Figure 4:**
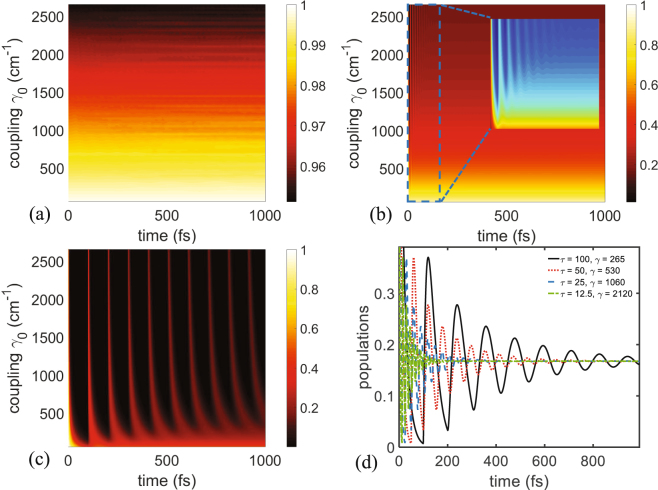
Effect of the coupling constant ${\gamma }_{0}$ on the population of the donor site for (**a**) $\tau =0.01$ fs, (**b**) $\tau =10$ fs, and (**c**) $\tau =100$ fs. [Note the difference in the scale in (**a**) in comparisons to (**b**) and (**c**)]. The inset of Fig. 4(b) shows a zoom of the population patterns to highlight short-lived quantum oscillations. (**d**) Figure showing the population of the dimer system for different values of $({\gamma }_{0},\tau )$. This shows how increasing $\tau$ (in units of femtoseconds) allows for lower values of the coupling coefficient to the environment (shown in units of cm^−1^).

To better illustrate the effect of environment feedback, the population of the donor site is given versus time and coupling constant ${\gamma }_{0}$ in the domains 1–1000 fs and 0–2500 cm^−1^, respectively. These plots shown in Fig. 4(a–c) correspond to different values of the time delay, i.e. 0.01 fs (almost no delay), 10 fs (moderate delay), and 100 fs (high delay), respectively. From the first two graphs (note the different scale of the color map of both these plots), it is obvious that no noticeable oscillations occur in the given domains. This confirms that when the delay is very small, no quantum oscillation can occur naturally, except when the value of the coupling is chosen to be very high. In contrast, Fig. 4(c) shows the same results for higher value of the delay (100 fs) and the oscillations can be observed for almost all values of ${\gamma }_{0}$. Figure 4(d) gives the population for coupled values of the delay time $\tau$ and coupling to the environment ${\gamma }_{0}$. These results further confirm that in order to observe quantum oscillations and long-lived coherence (few hundreds of femstoseconds) for moderate values of ${\gamma }_{0}$ (few hundreds of cm^−1^) one needs to tune the value of the delay accordingly. It should be also mentioned that in the case of small delay and high coupling (green dot-dashed curve of Fig. 4(d)) the oscillations die quicker than in the scenario of high delay and small coupling (black and red curves), even if the product $\tau {\gamma }_{0}$ is kept constant for both cases. This can be explained by the fact that the solution of the delay differential equation, i.e. Eq. (14) has its oscillations related to the delay $\tau$, so for higher values of $\tau$ the period is higher and the beats leave longer^44,45^.

## Discussion

In conclusion, we wish to emphasize the usefulness of taking into account the coherent feedback effect for modeling open quantum systems (e.g. a two-level system) interacting with a bosonic environment (e.g. a bath of phonons). In the simple example that we have considered in this work, i.e. a two-level system interacting with a phononic bath a two spatial locations, the role of delay was shown to be detrimental in the quantum beating and long-lived coherence. Although this model is very simple (described by Eq. (14)) one can gain from it considerable insight into the behavior of more complex system, e.g. the FMO molecules by extending it. In fact, we believe that such delay could have significant effect on the population quantum beatings of FMO systems, permitting thus to avoid unrealistically high values for the coupling to vibrational modes.
